# A SENIOR CENTER-BASED MULTILEVEL LIFESTYLE INTERVENTION IN OLDER ADULTS WITH TYPE 2 DIABETES

**DOI:** 10.1093/geroni/igae098.4280

**Published:** 2024-12-31

**Authors:** Yan Du, Lixin Song, Elena Volpi, Helen Hazuda, Zenong Yin, Erin Finley, Carlos Jaen, Josh Mireles

**Affiliations:** UT Health San Antonio, San Antonio, Texas, United States; University of Texas Health Science Center at San Antonio, San Antonio, Texas, United States; University of Texas Health San Antonio, San Antonio, Texas, United States; UT Health SA, San Antonio, Texas, United States; UTSA, San Antonio, Texas, United States; UT Health SA, San Antonio, Texas, United States; UT Health SA, San Antonio, Texas, United States; House of Neighbor Service, San Antonio, Texas, United States

## Abstract

Approximately 30% of adults aged 65 and over in the U.S. have Type 2 diabetes (T2D), leading to impaired physical function and reduced quality of life. This ongoing pilot project aims to assess the feasibility and acceptability of a multilevel lifestyle intervention at a day-activity senior center, examine preliminary effects, and explore implementation strategies. The study employs a 6-month, single-arm, pre- and post- test design (3 and 6 months). In collaboration with key stakeholders, including senior center staff and individuals with T2D, we are adapting the Diabetes Prevention Program curriculum and the Look AHEAD lifestyle intervention into a multilevel lifestyle intervention, targeting individual, interpersonal, and organizational levels. Nineteen older adults (mean age=68.5±5.5, Latinos/Hispanics=94.7%, female=89.5%) were enrolled. Fourteen digital lessons, facilitated by trained community health workers, are being provided separately in English and Spanish in a group format. Semi-structured interviews with key stakeholders will be conducted at 6 months to explore study acceptability and intervention adoption, implementation and sustainability. Baseline data showed that 83.3% of participants were obese, with 42.1% meeting physical activity recommendations. Self-efficacy scores for physical activity and diabetes management were 63.4±11.9 and 54.6±21.0. The mean HbA1c, extended short physical performance battery, and quality of life was 6.5±0.8, 15.7±3.8, and 40.8±7.8, respectively. The baseline data reveal significant challenges, such as high obesity rates and low self-efficacy in physical activity and diabetes management. We are leveraging these insights to tailor the multilevel lifestyle intervention to improve diabetes management, physical function, and quality of life in older adults with T2D.

